# Ozone Inhalation Attenuated the Effects of Budesonide on *Aspergillus fumigatus*-Induced Airway Inflammation and Hyperreactivity in Mice

**DOI:** 10.3389/fimmu.2019.02173

**Published:** 2019-09-13

**Authors:** Cameron H. Flayer, Moyar Q. Ge, Jin W. Hwang, Blerina Kokalari, Imre G. Redai, Zhilong Jiang, Angela Haczku

**Affiliations:** ^1^Department of Internal Medicine, University of California, Davis, Davis, CA, United States; ^2^Department of Internal Medicine, University of Pennsylvania, Philadelphia, PA, United States; ^3^Department of Pulmonary Medicine, Zhongshan Hospital, Fudan University, Shanghai, China

**Keywords:** asthma, allergy, ozone, budesonide, surfactant protein-D

## Abstract

Inhaled glucocorticoids form the mainstay of asthma treatment because of their anti-inflammatory effects in the lung. Exposure to the air pollutant ozone (O_3_) exacerbates chronic airways disease. We and others showed that presence of the epithelial-derived surfactant protein-D (SP-D) is important in immunoprotection against inflammatory changes including those induced by O_3_ inhalation in the airways. SP-D synthesis requires glucocorticoids. We hypothesized here that O_3_ exposure impairs glucocorticoid responsiveness (including SP-D production) in allergic airway inflammation. The effects of O_3_ inhalation and glucocorticoid treatment were studied in a mouse model of allergic asthma induced by sensitization and challenge with *Aspergillus fumigatus* (*Af*) *in vivo*. The role of O_3_ and glucocorticoids in regulation of SP-D expression was investigated in A549 and primary human type II alveolar epithelial cells *in vitro*. Budesonide inhibited airway hyperreactivity, eosinophil counts in the lung and bronchoalveolar lavage (BAL) and CCL11, IL-13, and IL-23p19 release in the BAL of mice sensitized and challenged with *Af* (*p* < 0.05). The inhibitory effects of budesonide were attenuated on inflammatory changes and were completely abolished on airway hyperreactivity after O_3_ exposure of mice sensitized and challenged with *Af*. O_3_ stimulated release of pro-neutrophilic mediators including CCL20 and IL-6 into the airways and impaired the inhibitory effects of budesonide on CCL11, IL-13 and IL-23. O_3_ also prevented budesonide-induced release of the immunoprotective lung collectin SP-D into the airways of allergen-challenged mice. O_3_ had a bi-phasic direct effect with early (<12 h) inhibition and late (>48 h) activation of SP-D mRNA (*sftpd*) *in vitro*. Dexamethasone and budesonide induced *sftpd* transcription and translation in human type II alveolar epithelial cells in a glucocorticoid receptor and STAT3 (an IL-6 responsive transcription factor) dependent manner. Our study indicates that O_3_ exposure counteracts the effects of budesonide on airway inflammation, airway hyperreactivity, and SP-D production. We speculate that impairment of SP-D expression may contribute to the acute O_3_-induced airway inflammation. Asthmatics exposed to high ambient O_3_ levels may become less responsive to glucocorticoid treatment during acute exacerbations.

## Introduction

In the era of novel biologicals being introduced into the clinic, glucocorticoids remain the main choice of asthma treatment due to their significant anti-inflammatory, immunosuppressive, and immunomodulatory effects (1, 2). A subset of patients however is refractory to glucocorticoids (3–7), making their asthma difficult to control (7). Glucocorticoid insensitivity can be a primary genetic trait but more commonly it is acquired during acute inflammatory exacerbations of airway disease (2, 4–6, 8). Epidemiologic studies indicate a causal link between air pollution and worldwide increases in asthma prevalence and severity. Inhalation of O_3_, an ubiquitous, oxidizing, and toxic air pollutant induces acute exacerbations with proinflammatory mediator release, neutrophilic granulocyte influx and obstruction of airways (9–15) and substantially worsens asthma morbidity and mortality (16, 17). Data obtained from studies on mice (18), dogs (19) rhesus macaques (20), healthy volunteers (21), and asthma patients (22, 23) have been controversial on whether glucocorticoids are effective to inhibit O_3_-induced exacerbation of airway inflammation and airway hyperreactivity in asthma. Further, the mechanisms of increased susceptibility of the asthmatic airways to O_3_ and how glucocorticoid action is affected by inhalation of this air pollutant remain unclear.

Individual susceptibility suggests that genetic predisposition is involved in O_3_ responsiveness (24). This is corroborated by strain dependence of the inflammatory response to O_3_ observed in mice (14, 15, 25). In addition, increasing evidence supports that a failure of protective immune mechanisms also likely plays an important role in shaping the O_3_ effects in the lung. Surfactant protein-D (SP-D), an epithelial cell product of the airways is a critical factor in the maintenance of pulmonary immune homeostasis. We have originally raised the importance of changes in SP-D expression in resolving allergen and O_3_-induced airway inflammation (26) by demonstrating that a differential ability of Balb/c and C57BL/6 mice to respond to allergen (27) or O_3_ (28), was inversely associated with the amount of SP-D recovered from the airways of these mouse strains (28, 29). Accordingly, genetically low SP-D producer or SP-D deficient mice were highly susceptible *to* and had a prolonged recovery period *from* airway inflammation after allergen or O_3_ exposure (28, 30, 31). O_3_-inhalation induced exacerbation of Th2-type airway inflammation in allergen challenged mice was also associated with the appearance of abnormal oligomeric molecular forms of SP-D indicating that oxidative damage can cause conformational change with a potential loss of its immunoprotective function (32, 33).

While our lab and others showed that glucocorticoids are necessary for expression of SP-D in epithelial cells (34–37), we also demonstrated a feedback regulation between SP-D and the Th2 cytokines IL-4/IL-13 (30) as well as IL-6 (28), respectively. Interestingly, we found no glucocorticoid response elements in the proximal promoter region of the SP-D gene (*sftpd*) however, this region contains an evolutionarily conserved STAT3/6 response element in a prominent proximal location. Pertinent to this, IL-4/IL-13 (activators of STAT6) as well as IL-6 (activator of STAT3) directly upregulated SP-D synthesis in airway epithelial cells *in vitro* and in mice *in vivo* (28, 30). Lastly, there are indications that STAT3 can be directly phosphorylated by H_2_O_2_ (the molecular product of O_3_ when mixed in water) treatment of airway epithelial cells *in vitro* (38).

We hypothesized that exposure to O_3_ interferes with the effects of glucocorticoids on *Af*-induced airway inflammation and hyperreactivity and, that O_3_ and glucocorticoid treatment have antagonistic effects on SP-D expression and function in the lung. To study these hypotheses we utilized our *in vivo* mouse model of combined *Af* + O_3_ exposure and *in vitro* human airway epithelial cell cultures.

## Materials and Methods

### *In vivo* Studies

**Figure 1:**
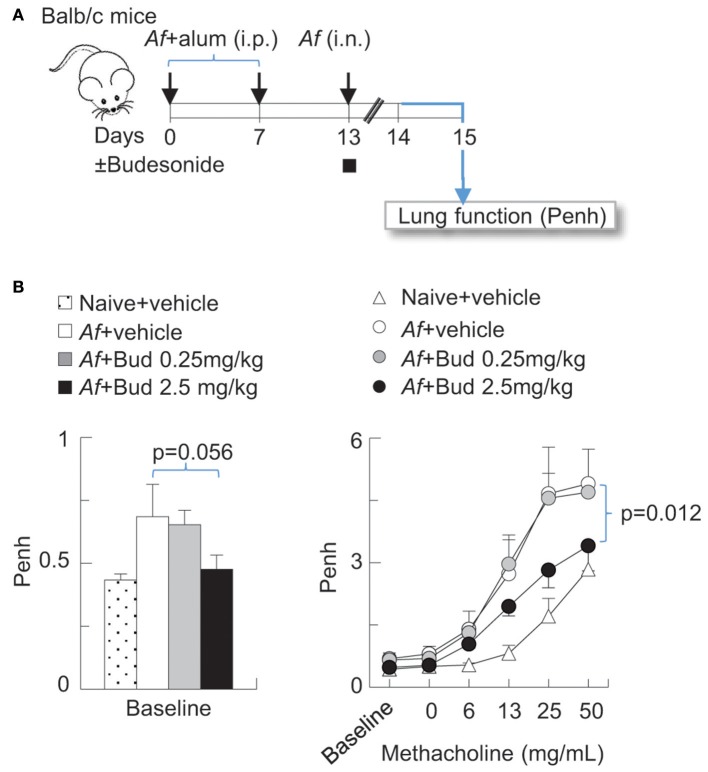
Budesonide inhibited airway hyperreactivity induced by *Af* sensitization and challenge in Balb/c mice, in a dose-dependent manner. **(A)** Balb/c mice were sensitized to 20 μg *Aspergillus fumigatus* (*Af*), with alum (i.p.) on days 0 and 7. On day 13, mice were challenged with 25 μg *Af* (i.n.) and administered 0 (DMSO vehicle only), 0.25 or 2.5 mg/kg budesonide. 48 h post-*Af* challenge mice were studied. **(B)** Lung function (enhanced pause, Penh) was measured by the Buxco^®^ system. Baseline measurements represent data collected over a 10 min period (left panel). Methacholine dose response was established to increasing concentrations of nebulized methacholine. Mean ± SEM of *n* = 4–18 (left panel: Dunett's multiple comparison; right panel: Two-way ANOVA with Tukey's multiple comparison).

Balb/c mice were obtained from the Jackson laboratories (Bar Harbor, ME) and bred in-house. All experiments were performed on 8–10 weeks old mice. Experiments where mice were sensitized and challenged with *Af* and exposed to air or O_3_ were carried out as previously described (30, 39, 40). In brief, mice were sensitized with 20 μg *Af* and alum by intraperitoneal injection (i.p.) on days 0 and 7, then challenged with 25 μg *Af* by intranasal (i.n.) instillation on day 13. In Figure 1, mice were treated with vehicle (Dimethyl sulfoxide, DMSO) or budesonide (0.25 or 2.5 mg/kg) i.n. at the time of *Af* challenge. 48 h post challenge, lung function (enhanced pause, Penh) was measured using the Buxco^®^ system.

**Figure 2:**
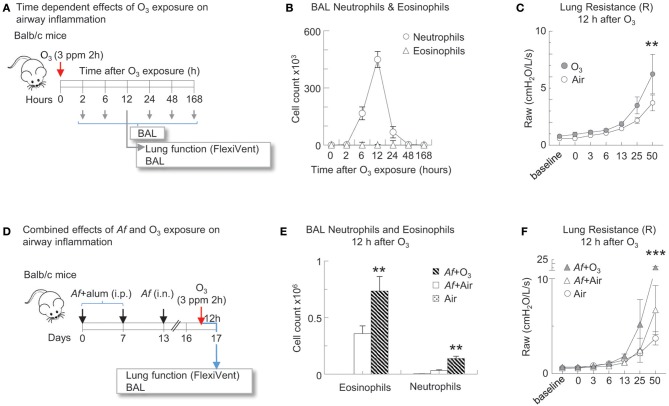
O_3_ induced airway inflammation and hyperreactivity and enhanced allergic airway changes in mice sensitized and challenged with *Af*. **(A)** Balb/c mice were exposed to air or 3 ppm O_3_ for 2 h and studied at the indicated time points for airway inflammation. 12 h after O_3_ exposure, lung function to methacholine was measured (Flexivent^®^) prior to BAL. **(B)** BAL neutrophils and eosinophils were quantified by differential cell counts on cytospin preparations multiplied by the total cell counts recovered from the BAL (Countess^®^). **(C)** O_3_ exposed mice and air exposed controls were studied for methacholine responsiveness 12 h later. Mean ± SEM of *n* = 6 ^**^p < 0.01 Two-way ANOVA with Tukey's multiple comparison's test (air vs. O_3_ exposure). **(D)** Balb/c mice were sensitized to 20 μg *Aspergillus fumigatus* (*Af*), with alum (*i.p*.) on days 0 and 7. On day 13, mice were challenged with 25 μg *Af* (*i.n*.). 82 h post-*Af* challenge, mice were exposed to air or 3 ppm O_3_ for 2 h, then 12 h later (96 h post-*Af* challenge), lung function was measured (Flexivent^®^) and BAL was harvested. **(E)** BAL neutrophils (live Ly6G^+^CD11b^+^ cells) and eosinophils (live CD11c^−^Siglec-F^+^ cells) were quantitated by FACS analysis. The absolute numbers of eosinophils and neutrophils were calculated by multiplying the percentage of cells determined by flow cytometric gating with the total numbers of cells/lung (Countess^®^). Mean ± SEM of *n* = 6; ^**^p < 0.01 Student's *t*-test (air vs. O_3_). **(F)** Lung function (airway resistance, Raw) was measured as indicated. Mean ± SEM of *n* = 6; ^***^p < 0.001 Two-way ANOVA with Tukey's multiple comparison's test (air vs. O_3_ exposure).

**Figure 3:**
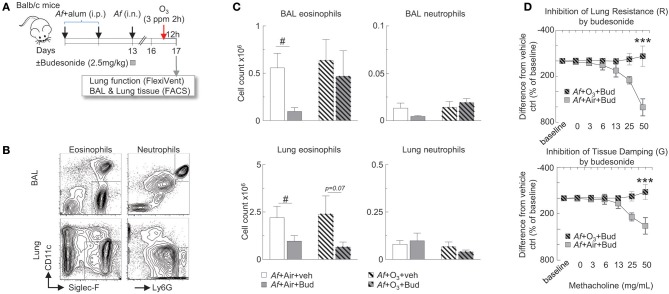
The inhibitory effects of budesonide on *Af*-induced airway inflammation were attenuated and on airway hyperreactivity were completely abolished by O_3_ exposure in sensitized and challenged mice. **(A)** Balb/c mice were sensitized to 20 μg *Aspergillus fumigatus* (*Af*), with alum (*i.p*.) on days 0 and 7. On day 13, mice were challenged with 25 μg *Af* (*i.n*.) and administered 2.5 mg/kg budesonide or vehicle (DMSO). 82 h post-*Af* challenge, mice were exposed to air or 3 ppm O_3_ for 2 h, then 12 h later (96 h post-*Af* challenge), lung function was measured (Flexivent^®^), and BAL and lungs were harvested. **(B)** Representative gating of BAL and lung neutrophils (live Ly6G^+^CD11b^+^ cells) and eosinophils (live CD11c^−^Siglec-F^+^ cells). **(C)** The absolute numbers of eosinophils and neutrophils were calculated by multiplying the percentage of cells determined by flow cytometric gating with the total numbers of cells/lung or BAL (Countess^®^). Mean ± SEM of *n* = 6; #*p* < 0.05 Student's *t*-test (vehicle vs. budesonide). **(D)** Lung resistance and tissue damping results were calculated as % change from baseline. The difference between the vehicle and budesonide treatment is depicted (by subtracting the individual % change from baseline values from the vehicle treatment average at each methacholine concentration). Mean ± SEM of *n* = 6–14; ^***^p < 0.001 (*Af* +Air+Bud vs. *Af* +O_3_+Bud) Two-way ANOVA with Tukey's multiple comparison's test.

**Figure 4:**
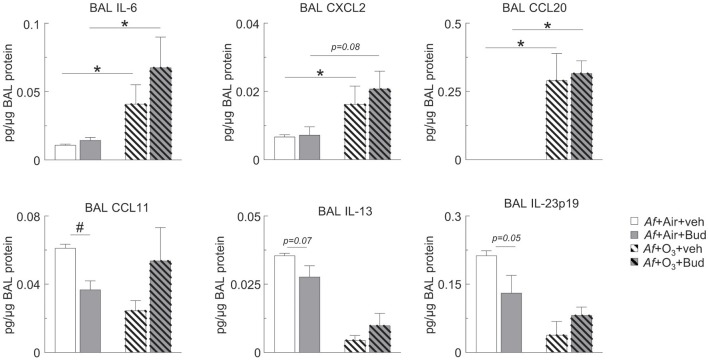
O_3_ upregulated BAL IL-6, CXCL2, and CCL20 in a budesonide-resistant manner and reversed the inhibitory effects of budesonide on CCL11, IL-13, and IL-23 expression in mice sensitized and challenged with *Af*. Cell-free BAL supernatant was assessed for the indicated cytokines and chemokines by a R&D mouse magnetic Luminex Assay (pg cytokine/μg BAL protein). Mean ± SEM of *n* = 3; ^*^p < 0.05, #*p* < 0.05 Student's *t*-test [vehicle vs. budesonide (#) or air vs. O_3_ (^*^)].

**Figure 5:**
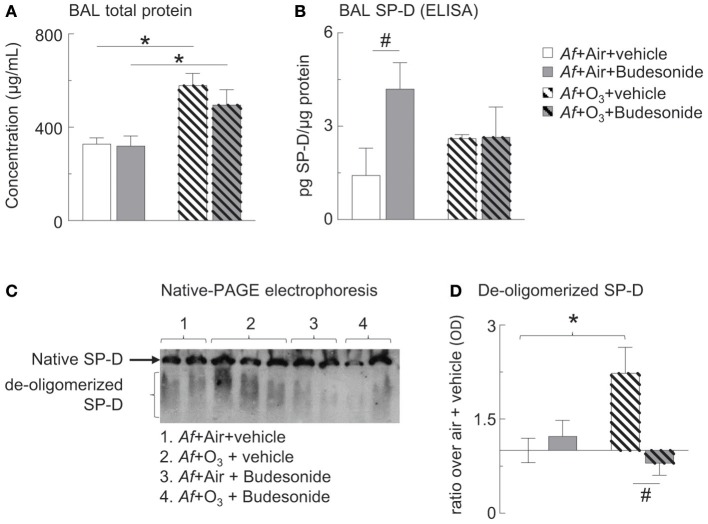
O_3_ inhibited budesonide-induced SP-D expression in *Af*-challenged mice and caused SP-D de-oligomerization in the BAL. **(A)** Cell-free BAL supernatant was assessed for total protein by BCA assay. **(B)** BAL SP-D was measured by ELISA. **(C)** Native-PAGE western blot was used to study SP-D structure. Native (intact) SP-D is the band that due to its molecular size remain on the top of the gel. Due to the variability of migratory capabilities of the de-oligomerized SP-D components, these appear as a “smear” throughout the gel. **(D)** SP-D optical density by Image J analysis; ratio over the mean value of “air+vehicle” control group data. Mean ± SEM or *n* = 5–6; ^*^p < 0.05 air vs. O_3_, #*p* < 0.05 vehicle vs. budesonide, Two-way ANOVA with Tukey's multiple comparison's test.

In Figures 2–5, mice followed the *Af* sensitization and challenge protocol as described, however 84 h post challenge/budesonide they were exposed to 3 ppm O_3_ or air for 2 h. Animals were studied 96 h post *Af* challenge (12 h post O_3_). These time points were selected to mimic O_3_-induced exacerbation of allergic changes, because by 96 h post *Af* challenge airway inflammation subsides while O_3_ exposure induced inflammation peaks 12 h post exposure (Figures 2A,B) (33, 40). That a 3 ppm inhaled dose in rodents results in O_3_ concentration in the lungs relevant to human exposure levels has been experimentally validated by others, using oxygen-18-labeled O_3_ (^18^O_3_). Hatch et al. showed that exposure to ^18^O_3_ (0.4 ppm for 2 h) caused 4–5-fold higher ^18^O_3_ concentrations in humans than in rats, in all of the BAL constituents measured (41). Rats exposed to 2.0 ppm, had still less ^18^O_3_ in BAL than humans exposed to 0.4 ppm. The species discrepancies between the recoverable O_3_ levels in the lung are not entirely clear. It is thought however that as rodents are obligate nose breathers (while humans breathe through their nose and mouth), this reduces the delivered dose of O_3_ to the lungs of rodents. Further, Slade et al. found that after exposure to O_3_, mice react by a rapid decrease of core temperature, a species and strain specific characteristics (42). The recoverable ^18^O_3_ in the lung tissue was negatively associated with the extent of hypothermia that significantly altered O_2_ consumption and pulmonary ventilation, explaining at least partly, the interspecies differences seen in O_3_ susceptibility. In addition, in pilot studies we also performed a careful assessment of the biological effects on a range of 0.5–6.0 ppm O_3_ exposure. Doses lower than 3 ppm did not elicit a significant inflammatory response that would be commensurate with what is seen in humans, in regards to BAL or peripheral blood neutrophilia, upon O_3_ inhalation for 2 h. Higher than 3 ppm doses caused observable respiratory distress especially in Balb/c mice. The O_3_ dose we used here therefore represents a level of exposure that is well-tolerated by both Balb/c and C56BL/6 mice and that causes a significant airway inflammatory response.

Lung function was measured using the Flexivent^®^ system (Scireq, Montreal, Canada) in response to increasing concentrations of inhaled methacholine. BAL and lung cells were harvested to study inflammatory cells by flow cytometry. Following collection of BAL, 10 mL ice-cold PBS was injected into the right ventricle to perfuse the lung. The lung lobes were then carefully removed and snipped into small pieces before undergoing digestion with Liberase TL (Millipore Sigma, Burlington, MA) for 40 min at 37°C on a shaker. Digested whole lung homogenate was filtered through a 70 μm cell strainer to create a single cell suspension for flow cytometric analysis. In cell-free BAL supernatant, a custom mouse magnetic Luminex assay was utilized to study cytokines and chemokines while SP-D was measured by western blot, native gel electrophoresis (structure) or sandwich ELISA (quantity). All mouse procedures were reviewed and approved by the University of California, Davis, and University of Pennsylvania Institutional Animal Care and Use Committees.

### Flow Cytometry

BAL and lung cells were harvested and single cell suspensions were prepared as previously described for analysis by flow cytometry (40). Fluorescently-conjugated monoclonal antibodies were purchased from Biolegend (San Diego, CA), BD Biosciences (San Jose, CA), or eBioscience (San Diego, CA). Single cell suspensions were incubated with antibodies targeting surface markers for 20 min at 4 degrees C in the dark. In the BAL samples, the following antibodies were used: APC-Cy7-CD11c, PE-Siglec F, PE-Cy7-CD11b, PerCP-Cy5.5-Ly6G. In the lung digest suspensions, neutrophils, and eosinophils were identified using APC-Cy7-CD11c, PE-Siglec F, and PerCP-Cy5.5-Ly6G. Live/dead Aqua was used in the panels throughout the study to exclude dead cells. Flow cytometry was performed on a Fortessa (BD Biosciences, San Jose, CA) and data was analyzed using FlowJo software (Ashland, OR).

### Luminex Assay

Cytokines and chemokines were assayed in the BAL via a custom Magnetic Mouse Luminex Assay (R&D System, Minneapolis, MN). C-C motif chemokine 11 (CCL11), Interleukin-23p19 (IL-23p19), IL-13, IL-6, Chemokine (CXC motif) ligand 2 (CXCL2), and CCL20 were measured in the premixed kit. BAL fluid was first concentrated using a 2 mL, 3 k Amicon Ultra Centrifugal Filter (Millipore Sigma, Burlington, MA) spun at 3,000 g for 30 min. The kit was performed following the instructions from the manufacturer.

### BAL SP-D Analysis

Total protein concentration was measured by the BCA Assay (Thermo Fisher Scientific, Waltham, MA). BAL SP-D was assayed by sandwich ELISA using our in-house generated monoclonal and polyclonal antibodies as previously described (33). BAL SP-D was also measured by native gel electrophoresis (33) to assess the tertiary structure of SP-D, which is critical to maintain its anti-inflammatory functions (43, 44). Proteins were transferred to a nitrocellulose membrane (Thermo Fisher Scientific, Waltham, MA). Goat anti-mouse SP-D (1:2,000, R&D Systems, Minneapolis, MN) was the primary antibody while donkey anti-goat antibody coupled to horseradish peroxidase (1:10,000 GE Healthcare Life Sciences, Marlborough, MA) was the secondary antibody. SP-D signal was detected using the ECL Western Blotting Substrate (Thermo Fisher Scientific, Waltham, MA) on film (ECL Hyperfilm, GE Healthcare Life Sciences, Marlborough, MA). Image J (National Institutes of Health, Rockville, MD) analysis was used to determine the optical density of SP-D bands.

### *In vitro* Studies

**Figure 6:**
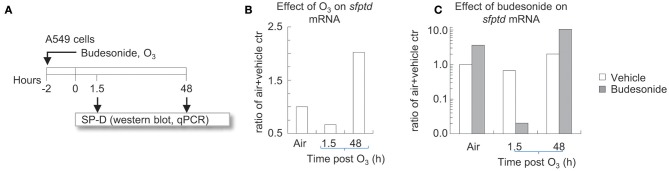
Time dependent effects of O_3_ on budesonide-induced *sftpd* mRNA in A549 cells *in vitro*. **(A)** A549 cells were incubated for 2 h with budesonide or vehicle (DMSO) and exposed to air or 300 ppb O_3_. 1.5 or 48 h after exposure/treatment ended, cells were harvested and processed for RNA extraction. **(B,C)** *sftpd* mRNA was measured by qPCR. Fold over air+vehicle values are shown. Representative of three independent experiments.

**Figure 7:**
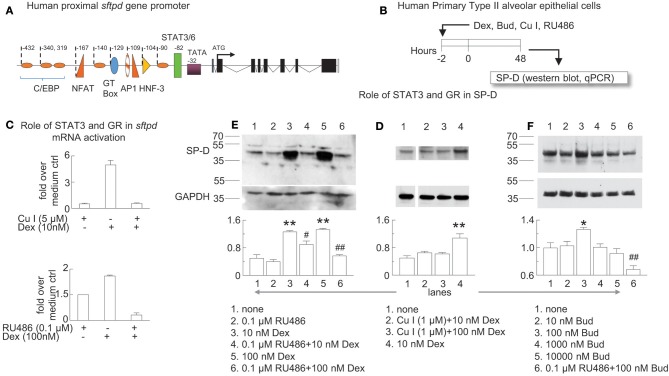
Glucocorticoid receptor-induced *sftpd* mRNA and protein expression is facilitated by STAT3/6 binding. **(A)** Schematic of the proximal promoter region of the human SP-D gene (*sftpd*) lacking glucocorticoid response elements. The approximate positions of C/EBP (orange ovals) and STAT3/6 (green rectangle) binding are depicted. Negative values indicate the number of base pairs relative to the start of transcription. **(B)** Human Primary Type II alveolar epithelial (hAECII) cells were incubated for 2 h with dexamethasone (Dex), curcurbitacin I (Cu I), and RU486 as indicated. 48 h later, cells were harvested and SP-D was studied by western blot (protein, relative expression to GAPDH) and qPCR (*sfptd* mRNA). **(C)** qPCR of *sftpd* (fold over no dexamethasone). **(D–F)** SP-D protein was studied by western blot, compared to control GAPDH. Optical density of SP-D and GAPDH by Image J analysis; GAPDH was subtracted from SP-D density then the ratio over the mean value of “no treatment” group was calculated. Mean ± SEM of *n* = 6 **(C)** or *n* = 3 **(D–F)**. ^*^p < 0.05, ^**^p < 0.01 vs. “no treatment”; ^#^*p* < 0.05, ^*##*^*p* < 0.01 vs. the same concentration of Dex or Bud alone; One-way ANOVA with Bonferroni's multiple comparison's test **(D–F)**. Western blots are representative of three independent experiments.

Human primary type II airway epithelial (hAECII) cells were acquired from normal human lung tissues from NDRI (National Disease Research Interchange). A549 cells were purchased from ATCC (Manassas, Virginia). Dexamethasone, budesonide, curcurbitacin I (Cu I), and RU486 was purchased from Millipore Sigma (Burlington, MA). A549 cells are a human type II alveolar epithelial cell line used by our laboratory and others (45–47) to model functions including expression of mRNA for SP-D. We used these readily available cells to establish conditions of SP-D mRNA expression upon treatment with ozone and budesonide (Figure 6). The budesonide effects were then recapitulated in primary hAECII cells (Figure 7). A549 cells were cultured in DMEM supplemented with 10% fetal bovine serum and 1% penicillin/streptomycin (Thermo Fisher Scientific, Waltham, MA). Primary hAECII cells were cultured in DMEM-H21 plus F-12 Ham's (1:1) supplied with 5% fetal bovine serum, 100 U/ml penicillin, 0.1 mg/ml streptomycin, 2 mM L-glutamine. D-valine (Invitrogen) prevented growth of fibroblasts. hAECII cells were treated with budesonide/dexamethasone for 2 h, with or without RU486 and Cu I added to the culture. A549 cells were treated with budesonide and exposed to air or O_3_ (300 ppb) for 2 h, which was generated as previously described (31). DMSO was used as the vehicle control in the *in vitro* studies. At the time points indicated in the figure, cells were harvested for analysis of *sftpd* mRNA (qPCR) or SP-D protein (western blot). Cells were harvested in TRIzol reagent (Thermo Fisher Scientific, Waltham, MA) for mRNA analysis by qPCR or RIPA buffer (Thermo Fisher Scientific, Waltham, MA) for protein analysis by western blot.

For the western blots, total intracellular protein was measured by the BCA assay, then 20 μg protein was loaded for each lane. The primary antibody was goat anti-SP-D (1:500). The secondary antibody was HRP anti-goat IgG (1:1,000). For control antibodies, the primary was rabbit anti-GAPDH (1:1,000) and the secondary was HRP anti-rabbit (1:5,000). All antibodies were purchased from Santa Cruz Biotechnology (Dallas, TX). Image J (National Institutes of Health, Rockville, MD) analysis was used to determine the optical density of SP-D bands. For the qPCR, RNA was extracted from the TRIzol by chloroform layering and isopropanol precipitation, then reverse transcribed into cDNA via the QuantiTect Reverse Transcription Kit (Qiagen, Hilden, Germany). qPCR was performed on the recovered cDNA using SYBR green reagents (Applied Biosystems, Foster City, CA) on a ViiA 7 Real-Time PCR system (Thermo Fisher Scientific, Waltham, MA). Fold change was calculated using the ΔΔCt method, first normalizing values to GAPDH. Human SP-D primer with sequence of 5′-ACACAGGCTGGTGGACAG-3′ (sense); 5′-TGTTGCAAGGCGGCATT-3′ (anti-sense) were used to produce 61 bp products.

### Statistical Analysis

All statistics were performed using Prism v7 software (GraphPad Inc., La Jolla, CA). Data are expressed as mean ± SEM and are representative of at least 2 independent experiments. A Student's *t*-test was used to compare vehicle vs. budesonide or air vs. O_3_. A One-way ANOVA with Tukey's multiple comparison's test was used in the budesonide dose response experiment (Figure 1). A Two-way ANOVA with Tukey's multiple comparison's test was used when comparing all experimental groups. A *p* < 0.05 was considered statistically significant.

## Results

### Budesonide Inhibited Airway Hyperreactivity Induced by *Af* Sensitization and Challenge in Balb/c Mice in a Dose-Dependent Manner

Inhaled glucocorticoids improve lung function and airway inflammation in allergic asthma (1, 48, 49) but their effectiveness in acute asthma exacerbations are subject of on-going investigations (49–51). We developed a model in which mice were sensitized (i.p.) and challenged with *Af* at the time of budesonide administration (i.n.) (Figure 1A). To establish the dose-dependent effects of budesonide on methacholine responsiveness we used Penh (enhanced pause), a non-invasive measure of airway obstruction, because it enabled us to obtain data from multiple individual animals simultaneously and complete a study using a large number of mice. These results showed that sensitization and challenge to *Af* significantly increased baseline Penh and methacholine responsiveness and that budesonide significantly inhibited airway hyperreactivity to methacholine at 2.5 mg/kg dose (Figure 1B). For the subsequent experiments presented in this paper we used this budesonide dose and confirmed its inhibitory effects on allergic airway hyperreactivity by the invasive FlexiVent^®^ system (Figures 2C,F, 3D). These data also provided the basis for the subsequent studies on the effects of O_3_ on allergic airway inflammation and glucocorticoid responsiveness.

### O_3_ Induced Airway Inflammation and Hyperreactivity and Enhanced Allergic Airway Changes in Mice Sensitized and Challenged With *Af*

To establish the time course of O_3_ induced airway inflammation, Balb/c mice were exposed to air or 3 ppm O_3_ for 2 h and studied at several time points afterwards (Figure 2A). Neutrophil influx into the airways peaked 12 h after O_3_ exposure (Figure 2B). O_3_ exposed mice and air exposed controls were studied for methacholine responsiveness at the 12 h time point. O_3_ induced a significant increase in methacholine responsiveness compared with air exposed controls (*p* < 0.01) (Figure 2C). To investigate the effects of O_3_ on allergic airway inflammation Balb/c mice were sensitized and challenged with *Af*. In this model the acute inflammatory changes resolve by 96 h after allergen challenge. We therefore studied the mice at this time point, but we also exposed them to either air or O_3_ 12 h before (Figure 2D). BAL neutrophils and eosinophils were quantitated by FACS analysis (the gating strategy is shown in Figure 3B). O_3_ exposure clearly enhanced numbers of eosinophils and neutrophils in the airways of *Af* sensitized and challenged mice in comparison with air exposure (*p* < 0.01; Figure 2E). In addition, lung resistance to methacholine challenge was also significantly amplified in the O_3_ exposed animals (*p* < 0.001; Figure 2F). These results confirm our previous findings (39) and strongly indicate that O_3_ induces airway hyperreactivity on its own and that it enhances airway changes in allergen sensitized and challenged animals.

### The Inhibitory Effects of Budesonide on *Af*-Induced Airway Inflammation Were Attenuated, and on Airway Hyperreactivity Were Completely Abolished by O_3_ Exposure in Sensitized and Challenged Mice

Experiments in dogs (19) our previous studies in healthy volunteers (52) and investigations in mild asthmatics (53) showed that glucocorticoid treatment inhibited O_3_-induced inflammation in the airways. However, how would O_3_ alter the inhibitory effects of budesonide on allergic airway inflammation and hyperreactivity has not been documented.

To study the hypothesis that O_3_ impairs the anti-inflammatory effects of budesonide, Balb/c mice were sensitized and challenged with *Af* as described, and were intranasally treated with 2.5 mg/kg budesonide or vehicle. 82 h post-*Af* challenge mice were exposed to air or 3 ppm O_3_ for 2 h, then 12 h later (96 h post-*Af* challenge), lung function was measured (Flexivent^®^), and BAL and lungs were harvested (Figure 3A). BAL eosinophils (live Siglec-F^+^CD11c^−^ cells) and neutrophils (live Ly6G^+^CD11b^+^ cells) and lung eosinophils (live CD45^+^CD11c^−^Siglec-F^+^) and neutrophils (live CD45^+^CD11c^−^Ly6G^+^) were analyzed by FACS from single cell suspensions as shown in Figure 3B. Budesonide significantly suppressed eosinophil (not neutrophil) numbers both in the BAL and the lung in air exposed but not in O_3_ exposed mice (Figure 3C). Strikingly, inhibition of lung resistance (upper panel) and tissue damping (lower panel) by budesonide seen in air exposed mice (gray plain squares) was completely abolished in O_3_-exposed mice (gray hatched squares, *p* < 0.001, Figure 3D). These data indicated that the inhibitory effects of budesonide on *Af*-induced airway inflammation were attenuated and on airway hyperreactivity were completely abolished by O_3_ exposure in sensitized and challenged mice.

### O_3_ Upregulated BAL IL-6, CXCL2 and CCL20 in a Budesonide-Resistant Manner and Reversed the Inhibitory Effects of Budesonide on CCL11, IL-13, and IL-23 Expression in Mice Sensitized and Challenged With *Af*

Since O_3_ mitigated the inhibitory effect of budesonide on airway eosinophilia and airway hyperreactivity, we wanted to investigate the underlying mediator profile. We measured BAL CCL11 (eotaxin, an eosinophil chemoattractant), IL-23p19, IL-6, CXCL2 (pro-neutrophilic mediators), CCL20 (a lymphocyte chemoattractant), and IL-13 (known to prime smooth muscle cells for airway hyperreactivity). O_3_ strongly induced BAL IL-6, CXCL2, and CCL20 in a budesonide-independent manner (Figure 4). Meanwhile, budesonide significantly reduced BAL CCL11 showed a trend for reduction of IL-13 (*p* = 0.07) and IL-23p19 (*p* = 0.05) in the BAL of air exposed, but not O_3_ exposed mice (Figure 4, lower panels**)**. These data suggested that O_3_ induced pro-neutrophilic factors regardless of budesonide treatment, and that the suppressive effects of budesonide on eosinophilia and airway hyperreactivity-inducing factors was attenuated by O_3._

### O_3_ Caused SP-D De-oligomerization and Inhibited Budesonide-Induced SP-D Expression in the BAL

We and others previously showed that SP-D plays an important role in suppressing proinflammatory mediator release in allergen or O_3_-induced airway inflammation (28–30, 54) and that production of SP-D required the presence of glucocorticoids in airway epithelium (34–37). Further, we found that O_3_-induced airway inflammation in allergen challenged mice resulted in abnormal oligomeric molecular forms of SP-D indicating that oxidative damage can cause conformational changes with a potential inactivation of SP-D's immunoprotective function (28, 32, 33). Here we wanted to investigate how the combination of allergen with O_3_ exposure would alter the glucocorticoid effects on SP-D expression.

Assessment of total BAL protein levels showed that those were returned to normal 96 h after *Af* challenge, indicating inflammatory resolution in air exposed mice. In O_3_ exposed mice however, BAL protein levels were significantly elevated indicating acute inflammation that was not prevented by budesonide treatment (Figure 5A). As expected on the basis of previous investigations (34–37) budesonide significantly increased BAL SP-D in air exposed mice. Importantly, this budesonide effect on SP-D expression was lost in O_3_ exposed mice (Figure 5B). By native gel electrophoresis, structurally intact SP-D was found at the top of the gel and did not separate from the well, while de-oligomerized SP-D was resolved as a smear (Figure 5C). Native SP-D density was not statistically different between the groups studied (Figure 5C). O_3_ caused de-oligomerization of SP-D in the BAL of mice sensitized and challenged with *Af*. This change was prevented by budesonide treatment (gray hatched bar, Figure 5D). Our data suggested that budesonide induction of SP-D is inhibited by O_3_. We speculate that in budesonide treated mice SP-D was indirectly protected from de-oligomerization possibly as a result of inhibition of eosinophils (the main source of iNOS and nitric oxide) in the lungs of mice (Figure 3C).

### Time Dependent Effects of O_3_ on Budesonide-Induced *sftpd* mRNA in A549 Cells *in vitro*

We previously showed that IL-6 directly induced SP-D in type II alveolar epithelial cell cultures (28). This is interesting in light of O_3_ while strongly inducing IL-6 (Figure 4), did not increase SP-D 12 h after exposure, but in fact it prevented the stimulatory effects of budesonide on SP-D release in the airways of mice (Figures 5A,B). To better understand the mechanisms of how budesonide and O_3_ regulate SP-D expression we used A549 cells, a readily available human type II alveolar epithelial cell line that models functions such as expression of the SP-D gene (*sftpd*, Figure 6A). To confirm our findings, the budesonide effects were then recapitulated in primary human type II alveolar epithelial cells (hAECII, Figure 7). O_3_ exposure of A549 cells inhibited *sftpd* expression 1.5 h later, but by 48 h post exposure this effect was reversed into an induction of the *sftpd* gene (Figure 6B). Budesonide induced *sftpd* mRNA in A549 cells exposed to air. O_3_ completely prevented the budesonide induction of the *sftpd* gene 1.5 h later. However, by 48 h O_3_ and budesonide synergistically increased *sftpd* mRNA (Figure 6C). These results are in line with our previous *in vivo* study on Balb/c mice (28) and suggest that O_3_ acts on sftpd transcription in a bi-phasic manner with an early phase inhibition (<12 h) and a late phase activation >48 h. Based on these and our previous findings on IL-6 we speculated that budesonide and O_3_ may interact on a common signaling pathway involved in SP-D transcription in airway epithelial cells.

### Glucocorticoid Receptor-Induced *sftpd* mRNA Transcription Is Facilitated by STAT3/6 Binding

The proximal promoter region of the human SP-D gene (*sftpd*) has binding elements for C/EBP, NFAT, AP1, HNF-3, and STAT3/6 that all contribute to transcription of SP-D (Figure 7A) (55). Dexamethasone induced lung SP-D in mice at the level of transcription in the absence of a full glucocorticoid response element in the proximal promoter region of *sftpd*. Zhang and colleagues previously reported that STAT3 can act as a co-activator in glucocorticoid receptor signaling (56). To test if the glucocorticoid receptor works in concert with the STAT3/6 binding element to induce SP-D we studied primary hAECII cells using a specific inhibitor of STAT3 (cucurbitacin Cu I) (57) and the glucocorticoid receptor (RU486). We treated human primary type II alveolar epithelial (hAECII) cells with budesonide and dexamethasone *in vitro* and studied SP-D mRNA (qPCR for *sftpd*) and protein (western blot for SP-D) expression (Figure 7B). We used dexamethasone in the *in vitro* experiments as a positive control because it is a well-characterized glucocorticoid that induces SP-D (35). Indeed, dexamethasone induced *sftpd* expression in human primary type II aleveolar epithelial cells that was abolished in the presence of Cu I or RU486 (Figure 7C). Similarly, Cu I and RU486 inhibited dexamethasone and budesonide-induced SP-D protein in hAECII cells (Figures 7D–F). Optical density analysis by Image J analysis confirmed that antagonism of the glucocorticoid receptor or STAT3 impaired dexamethasone-induced SP-D (Figures 7D,E) and that budesonide induced SP-D in a glucocorticoid receptor dependent manner (Figure 7F). These data suggested that glucocorticoid-induced SP-D synthesis was dependent on glucocorticoid receptor and STAT3 activation.

## Discussion

We report here the effects of O_3_ on intranasal budesonide treatment in allergic airway inflammation and hyperreactivity, implicate the alterations in SP-D expression in the O_3_-induced airway changes and propose the involvement of STAT3 in glucocorticoid signaling during *sftpd* transcription. Our study raises the significance of air pollution in the regulation of respiratory immunity and treatment responsiveness in asthma.

Inhaled glucocorticoids are currently the main choice for asthma treatment because they can profoundly improve lung function, alleviate airway inflammation and airway hyperreactivity (1, 48, 49) but their effectiveness in acute asthma exacerbations is subject of on-going debate (49–51, 58–60). Studies on asthma exacerbations caused by exposure to air pollutants are limited (61, 62) and the available experimental data on animals (18–20) and humans (21–23) are unclear on whether inhaled corticosteroids are effective to treat O_3_-induced airway inflammation and/or airway hyperreactivity in asthma. We wanted therefore to further investigate the effects of budesonide on O_3_-induced exacerbation of allergic airway changes. We found that in the *Af* sensitization and challenge model airway hyperreactivity to methacholine was inhibited by budesonide at 2.5 mg/kg dose. To mimic asthma exacerbation, we sensitized and challenged Balb/c mice with *Af*, waited for 4 days for the acute inflammation to subside and then exposed the mice to O_3_. Our results show that O_3_ exposure induced airway hyperreactivity on its own and significantly enhanced lung resistance to methacholine and the numbers of eosinophils and neutrophils in the airways of *Af* sensitized and challenged mice confirming previous findings (39). To study the hypothesis that O_3_ impairs the anti-inflammatory effects of budesonide, mice were intranasally treated with 2.5 mg/kg budesonide or vehicle. Budesonide significantly suppressed eosinophil (not neutrophil) numbers both in the BAL and the lung in air exposed but not in O_3_ exposed mice. Strikingly, inhibition of lung resistance by budesonide (seen in air exposed mice) was completely abolished by O_3_ exposure.

In various experimental conditions budesonide was previously shown to inhibit mediators relevant to O_3_-induced airway changes such as IL-6 (63), CCL11 (64), CXCL2 mRNA in the lung (65) and IL-13-induced *ex vivo* airway hyperreactivity (66), while CCL20 was actually stimulated by budesonide in asthmatic airway epithelial cells (67) and there is no data in the literature on the effects of budesonide on IL-23p19. O_3_ upregulated BAL IL-6, CXCL2, and CCL20 in a budesonide-resistant manner and reversed the inhibitory effects of budesonide on CCL11, IL-13, and IL-23p19 expression in mice sensitized and challenged with *Af*. Induction of CCL20 by O_3_ is interesting because CCL20 was thought to be responsible for recruitment of neutrophils into the airways conveying budesonide resistance (67). The role of CCL20 in O_3_-induced resistance to the budesonide effects however would need further confirmation. The reduction seen in BAL CCL11 and IL-23p19 of the budesonide treated air exposed animals corresponded with decreased BAL eosinophil and neutrophil counts, while reduced IL-13 matched the observed inhibition of airway hyperreactivity in the same animals. Since O_3_ exposure prevented these budesonide effects, it is possible that these mediators are directly involved in the immunologic and physiologic response to combined *Af* and O_3_ exposure. On the other hand, O_3_ induced IL-6, CXCL2, and CCL20 was not altered by budesonide treatment and thus may be implicated in the observed neutrophilic inflammation caused by O_3_ under allergic conditions. Our results are significant because they reproduce a glucocorticoid resistant airway inflammation and the hallmark characteristics of severe neutrophilic asthma exacerbation (68, 69).

SP-D plays an important role in suppressing proinflammatory mediator release in allergen or O_3_-induced airway inflammation (29, 30, 54). Levels of SP-D expression in the lung are correlated to disease severity in asthma (70, 71). Therapeutics that boost SP-D expression are thought to improve asthma symptoms (70–72). Indeed production of SP-D requires the presence of glucocorticoids in airway epithelium (34–37). Our previous work showed that O_3_ exposure induced the expression of SP-D in the BAL >48 h later, as a protective mechanism (28, 33, 39) but O_3_-induced airway inflammation in allergen challenged mice also led to appearance of abnormal oligomeric molecular forms of SP-D indicating that oxidative stress can cause conformational changes that can inactivate SP-D's immunoprotective function (28, 32, 33). Such de-oligomerization was due to S-nitrosylation of SH bonds responsible for holding the dodecameric SP-D together (43, 44, 73). S-nitrosylation of SP-D requires NO, resulted from increased iNOS activity produced by the large numbers of activated inflammatory cells, particularly eosinophils in the allergen and O_3_-exposed lung (33, 43, 44, 73). Here we wanted to know if the combination of allergen with O_3_ exposure would alter the glucocorticoid effects on SP-D expression and whether budesonide treatment would affect O_3_-induced SP-D de-oligomerization. As expected on the basis of previous investigations (34–37, 72) budesonide significantly increased BAL SP-D in air exposed mice. Importantly, this budesonide effect on SP-D expression was lost in O_3_ exposed mice. O_3_ in addition caused de-oligomerization of SP-D in the BAL of mice sensitized and challenged with *Af*. This change was prevented by budesonide treatment. We speculate that in budesonide treated mice SP-D was indirectly protected from de-oligomerization possibly as a result of inhibition of eosinophils (the main source of iNOS and nitric oxide) in the lungs of mice. Taken together, our data suggested that budesonide induction of SP-D is inhibited by O_3_ revealing a novel mechanism by which O_3_ antagonizes the therapeutic benefits of this inhaled glucocorticoid. We propose that budesonide enhances SP-D expression thereby amplifying its local therapeutic effects in asthma.

We previously showed that IL-6 directly induced SP-D in type II alveolar epithelial cell cultures (28). This is interesting in the light that O_3_ while strongly inducing IL-6, did not increase SP-D 12 h after exposure, but in fact it prevented the stimulatory effects of budesonide on SP-D release in the airways of mice. To better understand the mechanisms of how budesonide and O_3_ regulate SP-D expression we used A549 cells, a readily available human type II alveolar epithelial cell line that models functions such as expression of the SP-D gene (*sftpd*). To confirm our findings, the budesonide effects were then recapitulated in primary human type II alveolar epithelial cells. O_3_ exposure of A549 cells inhibited *sftpd* expression 1.5 h later, but by 48 h post exposure this effect was reversed into an induction of the *sftpd* gene. Budesonide induced *sftpd* mRNA in A549 cells exposed to air. O_3_ completely prevented the budesonide induction of the *sftpd* gene 1.5 h later. However, by 48 h O_3_ and budesonide synergistically increased *sftpd* mRNA. These results are in line with our previous *in vivo* study on Balb/c mice (28) and suggest that O_3_ acts on *sftpd* transcription in a bi-phasic manner with an early phase inhibition (<12 h) and a late phase activation >48 h. Based on these and our previous findings on IL-6 we speculated that budesonide and O_3_ may interact on a common signaling pathway involved in SP-D transcription in airway epithelial cells.

Two groups independently established that glucocorticoids induced SP-D mRNA protein *in vitro* and *in vivo* (34, 35). These pioneering studies showed that hydrocortisone and dexamethasone stimulated both *sftpd* mRNA and SP-D protein *in vitro* and *in vivo* in the fetal rat lung. Since the proximal promoter region of the SP-D gene does not contain complete binding elements for the glucocorticoid receptor, it was hypothesized that glucocorticoids indirectly induced expression of the *sftpd* gene or work in concert with other binding elements. The proximal promoter region of the human SP-D gene has binding elements for C/EBP, NFAT, AP1, HNF-3, and STAT3/6 that all contribute to transcription of SP-D (55). Interestingly, Zhang et al. reported that STAT3 (an IL-6 responsive transcription factor) can act as a co-activator in glucocorticoid receptor signaling (56) and H_2_O_2_-treatment directly phosphorylated STAT3 in airway epithelial cells (38). We tested the role of STAT3 and the glucocorticoid receptor in SP-D mRNA (*sftpd*) and protein expression. Dexamethasone induced *sftpd* expression in human primary type II aleveolar epithelial cells was abolished by blockade of either the glucocorticoid receptor or STAT3. We established here that dexamethasone induced *sftpd* mRNA and SP-D protein via the glucocorticoid receptor and critically, STAT3. Recent evidence suggests that O_3_-induced glucocorticoid insensitivity involves p38 MAPK, MKP-1, and IL-17A. Inhibition of p38 MAPK prevented the decreased the inhibitory effects of dexamethasone on O_3_ stimulated inflammation and IL-17A (18) and inhibition of IL-17A reduced dexamethasone insensitivity in a mouse model of chronic O_3_ exposure (74). Here we showed for the first time that STAT3 is involved in glucocorticoid-induced SP-D synthesis. Cooperation between the glucocorticoid receptor and STAT3 may be crucial for SP-D synthesis in airway epithelial cells.

There are likely many pathways that contribute to the BAL SP-D levels *in vivo*, including but not limited to budesonide treatment, O_3_ exposure, and BAL IL-6 expression. Since glucocorticoids are known to have numerous side effects and after chronic administration patients can become refractory, novel asthma therapeutics to induce SP-D may seek to directly activate STAT3 signaling (5).

While prior work suggested that O_3_ may impair the effectiveness of budesonide, here we studied the potential role for SP-D in this pathway. We propose a novel SP-D-mediated mechanism for the anti-inflammatory and functional effects of budesonide on the lung. A better understanding of how air pollutants such as O_3_ might affect asthma treatment will lead to improved therapeutic approaches.

## Ethics Statement

This study was carried out in accordance with the recommendations of the University of California, Davis and University of Pennsylvania Institutional Animal Care and Use Committees. The protocol was approved by the University of California, Davis, and University of Pennsylvania Institutional Animal Care and Use Committees.

## Author Contributions

CF conducted the *in vivo* SP-D and luminex experiments, assisted with flow cytometry and Flexivent analysis, and wrote the manuscript. MG conducted the *in vivo* and *in vitro* experiments (performing the Flexivent and flow cytometry experiments) and assisted with flow cytometry and Flexivent analysis. ZJ conducted the *in vitro* experiments (isolation, purification and culture of AECII cells, Western blot, and qRT-PCR analysis for SP-D expression in the cells after treatment). JH, BK, and IR assisted with *in vivo* experiments. AH lead all aspects of the study and edited the manuscript.

### Conflict of Interest Statement

The authors declare that the research was conducted in the absence of any commercial or financial relationships that could be construed as a potential conflict of interest.

## Abbreviations

O_3_: Ozone
SP-D: Surfactant protein-D
sftpd: Surfactant protein-D gene
Af: *Aspergillus fumigatus*
BAL: Bronchoalveolar lavage
AHR: Airway hyperreactivity
Human primary type II airway epithelial (hAECII) cells
Cu I: Curcurbitacin I
CCL-: C-C motif chemokine
IL-: Interleukin-
CXCL-: Chemokine (CXC motif) ligand
STAT3: Signal transducer and activator of transcription 3
qPCR, Quantitative: real-time polymerase chain reaction
ELISA: Enzyme-linked Immunosorbent assay
DMSO: Dimethyl sulfoxide
R: Lung Resistance
G: Tissue Damping
i. n.: Intranasal
i.p.: Intraperitoneal.
